# A Survey of Research Progress and Development Tendency of Attribute-Based Encryption

**DOI:** 10.1155/2014/193426

**Published:** 2014-07-02

**Authors:** Liaojun Pang, Jie Yang, Zhengtao Jiang

**Affiliations:** ^1^State Key Laboratory of Integrated Services Networks, Xidian University, Xi'an 710071, China; ^2^School of Life Science and Technology, Xidian University, Xi'an 710071, China; ^3^Department of Computer Science, Wayne State University, Detroit, MI 48202, USA; ^4^Department of Computer Science, Communication University of China, Beijing 100024, China

## Abstract

With the development of cryptography, the attribute-based encryption (ABE) draws widespread attention of the researchers in recent years. The ABE scheme, which belongs to the public key encryption mechanism, takes attributes as public key and associates them with the ciphertext or the user's secret key. It is an efficient way to solve open problems in access control scenarios, for example, how to provide data confidentiality and expressive access control at the same time. In this paper, we survey the basic ABE scheme and its two variants: the key-policy ABE (KP-ABE) scheme and the ciphertext-policy ABE (CP-ABE) scheme. We also pay attention to other researches relating to the ABE schemes, including multiauthority, user/attribute revocation, accountability, and proxy reencryption, with an extensive comparison of their functionality and performance. Finally, possible future works and some conclusions are pointed out.

## 1. Introduction

With the development of the Internet and the distributed computing technology, there is a growing demand for data sharing and processing in an open distributed computing environment. The data provider needs to provide expressive access control and data confidentiality when communicating with customers. What is more, it is urgent for large-scale distributed applications to support one-to-many communication mode to reduce the enormous costs of data encryption.

The traditional encryption mechanism based on public key infrastructure (PKI) [1] can achieve data confidentiality; however, it has disadvantages. On one hand, in order to encrypt data, the data provider needs firstly to obtain the public keys of authorized users and then sends the encrypted data separately to the corresponding user, which increases the processing overhead and the bandwidth demand [2]. On the other hand, although broadcast encryption [3] can solve the efficiency problem mentioned above, the data provider must obtain the user's list before encryption. In addition, if the data provider wants the recipient to be the one with certain identity not the one who is specified, the public key encryption will not work anymore. Therefore, more applicable encryption mechanisms are required.

Identity-based encryption (IBE) [4] mechanism allows a sender to encrypt a message to an identity without accessing his public key certificate, which simplifies the certificate management procedure and reduces certificate transmission overhead. The ability to carry out public key encryption without certificates makes IBE suitable for many practical applications. For example, Alice can send a message encrypted by Bob's email address (e.g., Bob@hotmail.com) to Bob without the support of PKI.

One common feature of all previous IBE schemes is that they regard identities as a string of characters. However, in 2005, Sahai and Waters [5] proposed a new type of IBE scheme called fuzzy IBE (FIBE) which regards identities as a set of descriptive attributes. FIBE can be regarded as the first concept of ABE in which the data owner can encrypt a message to all users that have a certain set of attributes. In the same year, Nali et al. [6] proposed a threshold ABE scheme. Although this scheme can prevent the collusion attacks, it introduces new disadvantage that the threshold semantics are limited in designing more general systems which need expressive access control.

In ABE scheme, attribute plays a very important role. Attributes have been exploited to generate a public key for encryption data and have been used as an access policy to control users' access. Based on the access policy, subsequent researches can be roughly categorized [7] as either key-policy or ciphertext-policy. The first KP-ABE scheme that allows any monotone access structures was proposed by Goyal et al. [7], and the first CP-ABE scheme was presented by Bethencourt et al. [8]. After that, several KP-ABE [9–11] and CP-ABE schemes [12–20] were proposed. Goyal et al. [12] presented a bounded CP-ABE scheme in the standard model, but the first fully expressive CP-ABE scheme in the standard model was proposed by Waters [13]. Subsequently, Attrapadung and Imai [21] proposed a Dual-Policy ABE scheme which allows key-policy and ciphertext-policy to act on encrypted data simultaneously.

Moreover, Müller et al. [22, 23] proposed a distributed ABE scheme with a constant number of bilinear pairing operations during decryption. Yu et al. [24] proposed a fine-grained data access control encryption scheme. Tang and Ji [25] proposed a verifiable ABE scheme, and Wang et al. [26, 27] proposed a hierarchical ABE (HABE) scheme in 2010 and 2011, respectively. In these schemes, Wang et al. used the disjunctive normal form policy to generate the keys hierarchically, assuming that all attributes in one conjunctive clause are administered by the same domain authority. More studies on HABE are in literatures [28–30].

In each ABE scheme mentioned above, the user must go to a trusted party to prove his identity before obtaining a secret key which allows him to decrypt messages. Chase [31] gave an efficient multiauthority ABE scheme in which the user's secret key is no longer authorized by a single center authority but authorized separately by different cooperative and independent authorities. In addition to this, there are also some multiauthority ABE schemes [31–37].

According to the existing schemes, a summary [38] of the criterial functionalities in an ideal ABE scheme is listed as follows. (1) Data confidentiality: unauthorized participants cannot know the information about the encrypted data. (2) Fine-grained access control: in order to achieve flexible access control, even for users in the same group, their access rights are not the same. (3) Scalability: the number of authorized users cannot affect the performance of the scheme. That is to say, the scheme can deal with the case that the number of the authorized users increases dynamically. (4) User/attribute revocation: if a user quits the system, the scheme can revoke his access right. Similarly, attribute revocation is inevitable. (5) Accountability: in all previous schemes, the dishonest users can just directly give away part of their original or transformed keys such that nobody can tell who has distributed these keys. The above problem which is called key abuse should be prevented by accountability. (6) Collusion resistance: the dishonest users cannot combine their attributes to decrypt the encrypted data.

In order to realize an ideal ABE scheme, some researches which are aimed at addressing the issue of user/attribute revocation [8, 9, 39–48] and accountability [49–53] in ABE schemes have been published on journals or academic conferences. What is more, with its own advantages, the attribute-based cryptosystem has the ability and possibility to be applied to other areas. Particularly, lots of studies which focus on the applications of ABE in proxy reencryption [54–59] have been proposed.

**Figure 1:**
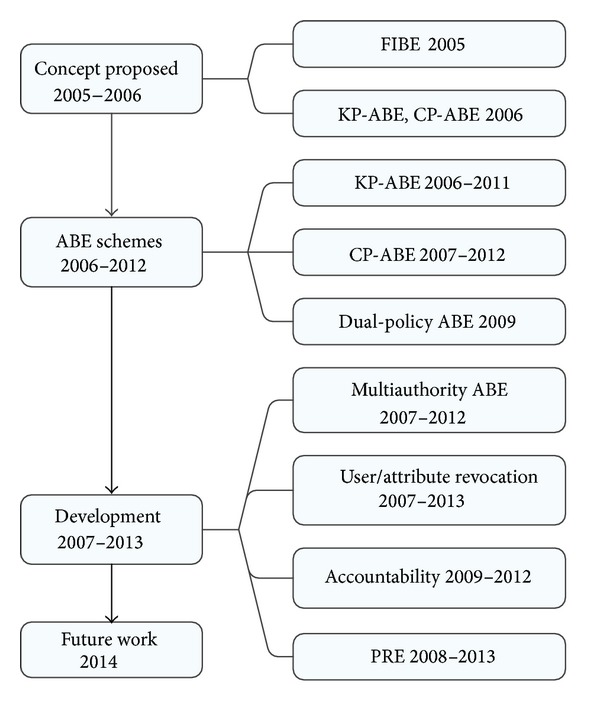
Development of ABE.

In conclusion, the existing research results about ABE can be generally divided into the design of ABE schemes, the multiauthority ABE schemes, and the user/attribute revocation, accountability, and applications of ABE schemes, which can be shown in Figure 1. According to this classification, the rest of this paper can be organized as follows. We introduce the basic ABE scheme in Section 2. The KP-ABE, CP-ABE, and Dual-policy ABE are examined in Section 3. Then, multiauthority ABE is surveyed in Section 4. User/attribute revocation and accountability in ABE are shown in Sections 5 and 6, respectively. One application of ABE, the attribute-based proxy reencryption, is surveyed in Section 7. What is more, in Section 8, we point out the problems worth further studying. Finally, we make some conclusions in Section 9.

## 2. Formal Model of the Basic ABE

In 2005, Sahai and Waters [5] proposed the FIBE which views identities as a set of descriptive attributes. With its basic and descriptive algorithms, to say the least, this scheme is usually regarded as the basic ABE scheme. In this section, firstly, we deal with the complexity assumptions used in the basic ABE scheme. Then, we give the formal algorithm and security model of it.

### 2.1. Complexity Assumptions

The complexity assumptions are stated below.

**Definition 1 (decisional bilinear Diffie-Hellman (BDH) assumption).**

Suppose a challenger chooses *a*, *b*, *c*, *z* ∈ *Z*_*p*_ at random. The decisional BDH assumption is that no polynomial-time adversary is able to distinguish the tuple (*A* = *g* ^*a*^, *B* = *g* ^*b*^, *C* = *g* ^*c*^, *Z* = *e*(*g*,*g*)^*abc*^) from the tuple (*A* = *g* ^*a*^, *B* = *g* ^*b*^, *C* = *g* ^*c*^, *Z* = *e*(*g*,*g*)^*z*^) with a negligible advantage.

**Definition 2 (decisional modified Bilinear Diffie-Hellman (MBDH) assumption).**

Suppose a challenger chooses *a*, *b*, *c*, *z* ∈ *Z*_*p*_ at random. The decisional MBDH assumption is that no polynomial-time adversary is able to distinguish the tuple (*A* = *g* ^*a*^, *B* = *g* ^*b*^, *C* = *g* ^*c*^, *Z* = *e*(*g*, *g*)^*ab*/*c*^) from (*A* = *g* ^*a*^, *B* = *g* ^*b*^, *C* = *g* ^*c*^, *Z* = *e*(*g*,*g*)^*z*^) with a negligible advantage.

### 2.2. Formal Definition of Algorithm Model

Sahai and Waters [5] gave the formal definition of the FIBE. Generally speaking, an ABE scheme usually consists of the following four fundamental algorithms, namely,* Setup*,* Key Generation*,* Encryption*, and* Decryption*, and it has a sender, an authority, and some receivers as participants.

The four algorithms in the basic ABE scheme are shown as follows.

*Setup.* This is a randomized algorithm performed by an authority in order to create a new ABE scheme. It takes no input other than the implicit security parameter *k* and outputs a set of public parameters PK and a master key MK.

*Key Generation.* The authority executes this algorithm for the purpose of generating a secret key. It takes as input a set of attributes *ω*, the master key MK, and the public parameters PK and outputs a decryption key SK.

*Encryption.* This randomized algorithm is run by a sender who wants to encrypt a message *m*, with a set of attributes *ω*′, and the public parameters PK. It outputs the ciphertext CT.

*Decryption.* This algorithm takes as input the ciphertext *E* that has been encrypted under the set *ω*′ of attributes, the decryption key SK associated with *ω*, and the public parameters PK. It outputs the message *M* if |*ω*∩*ω*′| ≥ *d*, and here *d* is a threshold parameter.

In the basic ABE scheme, the user's secret key and the ciphertext are labeled with sets of descriptive attributes. A particular key can decrypt a particular ciphertext only if there are at least *d* attributes overlapped between the attributes of the ciphertext and the user's key. The decryption condition in a KP-ABE or CP-ABE scheme is that the attributes set satisfies the access structure specified in the secret key or ciphertext.

### 2.3. Security Model

We now discuss the security of the basic ABE scheme. A selective-set model is defined for proving the security of the scheme under chosen plaintext attack [5]. The fuzzy selective-ID game is very similar to the standard selective-ID model for identity-based encryption [4] with the exception that the adversary is only allowed to query for secret keys for identities which have *d* − 1 or less attributes overlapped with the target identify.

The selective-ID game played between a challenger and an adversary is shown below.

*Fuzzy Selective-ID Model of the Basic ABE*

*Init.* The adversary declares the identity, *α*, upon which he wishes to be challenged.

*Setup.* The challenger runs the* Setup* algorithm and tells the adversary the public parameters. 

*Phase 1.* The adversary is allowed to issue queries for secret keys of multiple identities, *γ*_*j*_, where |*γ*_*j*_∩*α*| < *d*, for all *j*. 

*Challenge.* The adversary submits two messages *M*_0_ and *M*_1_ with equal length. The challenger flips a random coin to choose a value *b* and encrypts *M*_*b*_ with *α*. The ciphertext is passed to the adversary. 

*Phase 2.* Phase  1 is repeated. 

*Guess.* The adversary outputs a guess *b*′ of *b*.

The advantage of an adversary *A* in this game is defined as

(1) $$\begin{array}{c} \text{Ad}{\text{v}}_{A}=\left|\text{pr}\left[b^{\prime }=b\right]-\frac{1}{2}\right|. \end{array}$$

**Definition 3 .**

A scheme is secure in the fuzzy selective-ID security model if all polynomial-time adversaries have at most a negligible advantage in the above game.

Sahai and Waters [5] proved the CPA security of the basic ABE scheme in the selective-ID model by reducing it to the hardness of the decisional MBDH assumption. They also pointed out that the scheme can be extended to the chosen-ciphertext model by applying the technique of the simulation-sound noninteractive zero knowledge (NIZK) proofs to achieve the CCA security [60]. It is well known that the CPA security is the most basic security requirement of the public key encryption mechanism and that the CCA security is a stronger one. However, most of the existing ABE schemes can only be proved CPA secure and it still remains as an open problem to design a CCA secure ABE scheme. To some degree, the security proofs in the existing ABE schemes have the same thought with the idea that an ABE scheme is a secure one if no probabilistic polynomial time adversary *A* can win the corresponding game with a nonnegligible advantage, a generally accepted fact that will be shown in the next section.

## 3. ABE Schemes

With stronger and richer expression capability, the FIBE [5] scheme which was introduced in Section 2 is considered as the extension of the traditional IBE scheme [4]. In an FIBE scheme, ciphertexts are labeled with a set of attributes *ω* and a user's secret key is associated with both a threshold parameter *d* and another set of attributes *ω*′. To enable a user to decrypt a ciphertext, it is inevitable that there are at least *d* attributes overlapped between the ciphertext and his secret key. The only access structure supported in the FIBE scheme is “*threshold*” which is fixed at the setup phase by the authority. However, there is an increasing need of flexible access control policies supporting the operations like “*and*,” “*or*,” “*threshold*,” “*non,*” and so forth, in many practical applications. That is to say, the FIBE scheme is limited in many general application scenarios. Therefore, more and richer types of ABE schemes were proposed. These schemes in accordance with the different protection strategy deployment ways can be divided into two main categories [7]: KP-ABE schemes and CP-ABE schemes. Also, there is a hybrid type called the dual-policy ABE scheme, a combination of the above two types. A brief introduction to these schemes will be given in this section.

### 3.1. KP-ABE

In 2006, Goyal et al. [7] introduced the idea of a more general key-policy attribute-based cryptosystem for fine-grained sharing of encrypted data and proved its security in the attribute-based selective-set model under the decisional bilinear Diffie-Hellman (DBDH) assumption. This scheme is called the KP-ABE scheme since each secret key is associated with a tree access structure which specifies the type of ciphertexts which can be decrypted by this secret key, where ciphertexts are simply labeled with a set of descriptive attributes. If and only if the attributes set satisfies the access structure specified in the secret key, the user can decrypt the ciphertext. Their scheme gives us a powerful tool for encryption with fine-grained access control for applications such as sharing audit log information. It also supports delegation of secret keys. Unfortunately, with a drawback that the access policy is built into the secret key, the data owner in a KP-ABE scheme cannot decide the one who can decrypt the ciphertext, and he can only choose a set of attributes to control the access of ciphertexts. Besides, the access structure is a monotonic access structure which cannot express the negative attribute to exclude the participants with whom the data owner does not want to share data.

Subsequently, Ostrovsky et al. [9] proposed a scheme with a nonmonotonic access structure where the secret keys are labeled with a set of attributes including positive and negative attributes. Comparatively, the ABE scheme with nonmonotonic access structure can express a more complicated access policy. Unfortunately, this mechanism doubles the size of the ciphertext and secret key and adds encryption/decryption overheads at the same time. Ostrovsky et al.'s initial construction is recently improved by Lewko et al. [10] who used a new technique to achieve user revocation and design the most efficient nonmonotonic KP-ABE scheme.

In the above KP-ABE schemes, the ciphertext size grows linearly with the number of ciphertext attributes and the only known exception only supports restricted forms of threshold access policies. Attrapadung et al. [11] proposed the first KP-ABE scheme with nonmonotonic access structures and constant ciphertext size. The disadvantage is that the secret key has quadratic size in the number of attributes.

### 3.2. CP-ABE

Goyal et al. [7] suggested the possibility of a CP-ABE scheme, but they did not offer any constructions. In a CP-ABE scheme, a user's secret key will be associated with an arbitrary number of attributes expressed as strings, while ciphertext is associated with an access structure. A user will only be able to decrypt a ciphertext if his attributes satisfy the access structure of the ciphertext.

In 2007, using a monotonic access tree as access structure, Bethencourt et al. [8] proposed the first CP-ABE construction. Their scheme can support flexible access control policies like the KP-ABE [7] scheme, but the security proof is in the generic group model.

Cheung and Newport [14] provided a provably secure CP-ABE scheme which is proved to be secure under the standard model and their scheme supports AND gate on positive and negative attributes as its access policy. They use a* do not care* element to indicate the attribute which does not appear in the AND gate. Intuitively, the public key elements *T*_*i*_, *T*_*n*+*i*_, and *T*_2*n*+*i*_ correspond to the three types of occurrences of *i*: positive, negative, and* do not care*. This scheme is proved to be the CPA secure under the DBDH assumption for the first time. And, it improves the security proof in Bethencourt et al.'s [8]. Unfortunately, two drawbacks remain. Firstly, it is not sufficiently expressive because it supports only policies with logical conjunction. Secondly, the size of the ciphertext and the secret key increases linearly with the total number of attributes in this scheme. These two shortcomings make this scheme less efficient than Bethencourt et al.'s [8].

Based on Cheung and Newport's scheme [14], Nishide et al. [15] and Emura et al. [16] improved the efficiency and achieved hidden policies, respectively. Nishide et al. [15] proposed a scheme with AND gates on multi-value attributes as its access policy. Emura et al. [16] used the same access policy and propose an improved scheme. And this scheme also achieves a constant length of ciphertext and constant number of bilinear pairing operations.

In order to design CP-ABE scheme with flexible strategy under the DBDH assumption, Goyal et al. [12] and Liang et al. [17] adopted bounded tree structure. Goyal et al. [12] presented a bounded CP-ABE (BCP-ABE) scheme in the standard model and generalized the transformational approach to show how to transform a KP-ABE scheme into a CP-ABE one by using what they called “universal access tree.” The BCP-ABE scheme supports any access formulas of polynomial bounded size (including the “*and*,” “*or*,” and “*threshold*” operations) with a shortcoming that the sender is restricted to use only an access tree whose depth *d*′ ≤ *d* (here *d* indicates the depth of the access trees defined in the setup phase). Liang et al. [17] improved the BCP-ABE scheme [12] by improving the efficiency of the encryption/decryption algorithm and shortening the length of public key, secret key, and ciphertext.

Later, Ibraimi et al. [18] used the general access tree structure to eliminate the boundary constraints in [12, 17] and presented a new technique to realize the CP-ABE scheme without Shamir's threshold secret sharing. In their scheme, the sender defines the privacy policy by using an access tree which is *n*-ary tree represented by* and* and* or* nodes. Note that, realizing a scheme without threshold secret sharing is important for resource-constrained devices since calculating polynomial interpolations to construct the secret is computationally expensive. Finally, compared with Cheung and Newport's [14], it requires less computation overheads during the* Encryption*,* Key Generation*, and* Decryption phases*.

In 2011, Waters [13] proposed a new methodology for realizing CP-ABE under concrete and noninteractive cryptographic assumptions in the standard model. He expressed access control by a linear secret sharing scheme (LSSS) matrix *M* over the attributes in the system (previously used structures can be expressed succinctly in terms of an LSSS). In this most efficient scheme, the ciphertext size and the encryption/decryption overheads increase linearly with the complexity of the access formula. As a result, his scheme achieves the same performance and functionality as Bethencourt et al.'s [8].

Finally, Lewko et al. [19] recently leveraged the encoding technique from Waters's scheme [13] to propose an ABE scheme that achieves adaptive (nonselective) security. Their scheme is based on composite order groups, which results in some loss of practical efficiency when compared with Waters'.

In recent years, almost all the schemes available, to the best of our knowledge, are constructed from bilinear pairings. J. Zhang and Z. F. Zhang [20] presented a CP-ABE scheme which supports AND gates without bilinear pairings. Their scheme is built based on *q*-ary lattices and has a very strong security proof based on worst-case hardness. Though it seems to be not much efficient, it gives light to the possibility of constructing attribute-based schemes under other hard problem assumptions (i.e., lattice problems), instead of the bilinear pairing-related assumptions.

### 3.3. Dual-Policy ABE

In 2009, Attrapadung and Imai [21] presented a new ABE scheme called the Dual-Policy ABE. Basically, it is a conjunctively combined scheme of Goyal et al.'s KP-ABE scheme [7] and Waters' CP-ABE scheme [13]. It allows simultaneously two access control mechanisms over encrypted data. One involves policies over objective attributes ascribed to data and the other involves policies over subjective attributes ascribed to user credentials. These two access control mechanisms can only allow either functionality above one at a time. What is more, the security proof is based on decisional bilinear Diffie-Hellman exponent (DBDHE) assumption.

### 3.4. Comparison

From what has been mentioned above, it is obvious that the basic ABE scheme and KP-ABE and CP-ABE schemes are different in complexity hypothesis, strategic flexibility, and applications. A conclusion can be made as follows.

The basic ABE scheme, which only supports “*threshold*” policy, is suitable for simply policy-required applications. At the same time, KP-ABE and CP-ABE schemes, which support complex strategies, are appropriate for the applications of fine-grained data sharing. In addition, in KP-ABE schemes, the access policy is built into the user's secret key, so the data owner cannot choose the person who can decrypt the data. Compared with KP-ABE schemes, CP-ABE schemes are more suitable for the realistic scenes. Generally speaking, KP-ABE schemes apply to query applications, such as pay TV system, audit log, targeted broadcast, and database access. On the contrary, CP-ABE schemes are used for access control applications, such as social networking site access, and electronic medical system.

The security model of the basic ABE scheme has been shown in Section 2. Both the basic ABE scheme and KP-ABE schemes [7, 9] use the DBDH assumption. And the situation in CP-ABE schemes is more complex. It is known that the more complex a strategy is, the more complex a CP-ABE scheme will be and the more difficult it is to prove its security. To achieve the CPA security under the standard complexity assumption, the main research on the CP-ABE is focused on designing the access structure. According to different access structures, the research can be divided into three kinds: AND gate, Tree, and LSSS matrix. Now a comparison of* Access structure*,* Complexity assumption*,* Security model*, and* Supported policy* in different CP-ABE schemes is made in Table 1.

The comparisons of the size of keys and ciphertext and the encryption/decryption computation overhead in different CP-ABE schemes are given in Tables 2 and 3, respectively. We can draw a conclusion from these tables: Emura et al.'s [16] scheme is the shortest in ciphertext and SK, Bethencourt et al.'s [8] in PK, and Waters' [13] in MK. What is more, in Bethencourt et al.'s [8], PK and MK have nothing to do with system attributes. As for computation overhead, Emura et al.'s [16] processes the lowest encryption/decryption overhead, and Ibraimi et al.'s [18] scheme has a lower one than Waters' [13].

## 4. Multiauthority ABE

Sahai and Waters [5] introduced a single-authority ABE scheme; however, they left the following open question: is it possible to construct an ABE scheme in which multiple authorities operate simultaneously, each distributing secret subkeys for a different set of attributes during the* Key Generation* phase? Subsequently, this question was answered by Chase [31] who proposed the first multiauthority ABE scheme.

In a single-authority ABE scheme, the authority can decrypt all ciphertexts, which is not proper from the point of security. Therefore, multiauthority ABE schemes [31–37] were proposed. These schemes can be divided into two types. One needs a central authority (CA, for short) which is used to guarantee the proper decryption and can also decrypt all ciphertexts, such as schemes [31, 33, 36], while the other does not need a CA, such as schemes [32, 34, 35, 37]. In this section, we survey these existing multiauthority ABE schemes in detail.

### 4.1. Multiauthority ABE with a CA

Chase's [31] proposed the first multiauthority ABE scheme where there are one central authority and *N* attribute authorities. The CA issues identity-related keys to users and the attribute authorities manage attributes and issue attribute-related keys. A user's keys from different attribute authorities are linked together by the user's global identifier (GID). In Chase's scheme, an sender specifies, for each attribute authority {*j*}_1≤*j*≤*N*_, a set of attributes and a trapdoor value *d*_*j*_. He can then encrypt a message such that a user can only decrypt if he has at least *d*_*j*_ of the given attributes from each attribute authority *j*. Although this scheme increases the computation and communication cost and needs to maintain such a fully trusted authority, Chase made an important step from the single-authority ABE to the multiauthority ABE.

To solve the problem that the CA must be fully trusted in Chase's [31] scheme, Bozovic et al. [33] constructed a threshold multiauthority ABE scheme which offers the same security guarantees provided by Chase. In addition, it can tolerate an “honest-but-curious” CA which has a definition that it honestly follows the protocol, while it is curious to decrypt arbitrary ciphertexts, thus violating the intent of the encrypting party.

Recently, based on Lewko et al.'s CP-ABE scheme [19], Liu et al. [36] proposed an adaptive secure multiauthority CP-ABE scheme which has multiple central authorities and attribute authorities in the standard model. The central authorities issue identity-related keys to users and the attribute authorities issue attribute-related keys to users. Prior to obtaining attribute keys from the attribute authorities, the user must obtain his secret keys from multiple central authorities. In terms of efficiency, this scheme is the same with Lewko et al.'s [19].

### 4.2. Multiauthority ABE without a CA

The utilization of a CA brings new security vulnerability and increases the computation and communication cost. So, in 2010, Lin et al. [32] adopted the distributed key generation (DKG) protocol [60] and the joint zero secret sharing (JZSS) [61] protocol to construct the secure threshold multiauthority fuzzy identity-based encryption (threshold MA-FIBE) scheme without a central authority for the first time. To initialize the idea, the multiple authorities must cooperatively execute the DKG protocol and the JZSS protocol twice and *k* times, respectively, where *k* is the degree of the polynomial selected by each authority. Each authority must maintain *k* + 2 secret keys. This scheme is *k*-resilient; namely, the scheme is secure if and only if the number of the colluding users is no more than *k*, and *k* must be fixed in the setup algorithm.

Chase and Chow [34] proposed a multiauthority KP-ABE scheme which removes the central authority by using a distributed PRF (pseudorandom functions) technique. Notably, they also addressed the privacy of the user. In previous multiauthority ABE schemes [31, 32], the user must submit his GID to each authority to obtain the corresponding secret key. This will increase the risk of user traced by a group of corrupted authorities. In order to avoid this risk, Chase and Chow [34] provided an anonymous key issuing protocol for the GID, where a 2-party secure computation technique is employed. This scheme is (*N* − 2)-tolerant; namely, the scheme is secure if and only if the number of the corrupted authorities is no more than *N* − 2, where *N* is the number of the authorities. Chase and Chow also left an open problem on how to construct a privacy preserving multiauthority ABE scheme without the need of cooperation among the authorities.

Han et al. [37] answered the question left by Chase and Chow [34] affirmatively by proposing a decentralized KP-ABE scheme with the privacy-preserving key extraction protocol. In their scheme, multiple authorities can work independently without any cooperation and a central authority. The GID is used to tie all the user's secret keys together, while the corrupted authorities cannot pool the user's attributes by tracing it. The scheme is any number tolerant for the users and (*N* − 1)-tolerant for the authorities, where *N* is the number of the authorities.

In 2011, Lekwo and Waters [35] proposed a new multiauthority scheme. Although their scheme may become inefficient for large attribute universe [13], it is the first adaptively secure multiauthority CP-ABE scheme proved in the random oracle model. This scheme improves the previous multiauthority ABE schemes, because it does not require collaboration among multiple authorities in the setup and key generation phases, and there is no central authority. Note that the authority in this scheme can join or leave the system freely without reinitializing the system. Besides the low efficiency, this scheme has another drawback that the attributes of the user can be collected by tracing his GID.

### 4.3. Comparison

The comparison between the different multiauthority schemes is shown in Tables 4 and 5. By |*U*|, |*A*_*U*_|, and |*A*_*C*_|, we denote the number of the universal attributes, the attributes held by user *U*, and the attributes required by the ciphertext, respectively. *I*_*U*_ and *I*_*C*_ denote the index set of the authorities. By *E* and *P*, we denote one exponential and one paring operation, respectively. By *L*_*G*_1__ and *L*_*G*_2__, we denote one element in group *G*_1_ and one element in group *G*_2_, respectively. *N* denotes the number of the authorities in the systems. By *d*, we denote the number of the central authorities in [36].

## 5. Revocation Mechanism of ABE

Revocation mechanism is necessary for any multiuser encryption systems to deal with malicious behaviors. The revocation mechanism of ABE schemes is more complicated than that of traditional public key cryptosystem or IBE schemes [40, 62–65]. For example, in CP-ABE schemes, different users may hold the same secret key in function related to the same attribute set, leading to additional difficulties in the design of a revocation mechanism.

In this section, we focus on ABE schemes that support revocation. In attribute-based setting, revocation mechanism can usually be divided into two kinds: user revocation and attribute revocation. Currently, there are mainly two ways to realize revocation [48]: one is the indirect revocation method [8, 39–44] and the other is the direct revocation method [9, 45–47].

### 5.1. Indirect Revocation Method

The indirect revocation method enforces revocation by the authority who releases a key update material periodically in such a way that only nonrevoked users can update their keys (hence, revoked users' keys are implicitly rendered useless). The indirect method has an advantage that senders do not need to know the revocation list. However, it also has a disadvantage that the key update phase can be a bottleneck since it requires communication from the authority to all nonrevoked users at all time slots. Recently, several attribute revocable ABE schemes have been proposed based on the indirect revocation method [8, 39–44].

There are several schemes [8, 39, 40] which realize attribute revocation by setting expiration time on each attribute. However, these approaches have two main problems. One is the security degradation in terms of the backward and forward security [43]. The other is the scalability problem. The authority periodically announces a key update material at each time slot so that all of the nonrevoked users can update their keys, which leads to a bottleneck for the authority.

To reduce the burden of authority and achieve immediate attribute revocation, two CP-ABE schemes with immediate attribute revocation with the help of semihonest service provider were proposed by Ibraimi et al. [41] and Yu et al. [42], respectively. However, they also have failed to achieve fine-grained user access control in the data outsourcing environment.

For this reason, Hur and Noh [43] proposed a CP-ABE scheme with fine-grained attribute revocation with the help of the honest-but-curious proxy deployed in the data service provider. It is an efficient revocation method by employing the binary tree representing revocation introduced by Boldyreva et al. [40] and reencrypting the ciphertext. However, their scheme cannot resist the collusion attack.

Aiming at reducing the computation overhead of data service manager, Xie et al. [44] proposed new CP-ABE construction with efficient user and attribute revocation. Compared with Hur and Noh's [43], in the key update phase, the computation overhead of the data service manager will be reduced by half.

### 5.2. Direct Revocation Method

The direct revocation method enforces revocation directly by the sender who specifies the revocation list while encrypting the ciphertext. An advantage of the direct method over the indirect one is that it does not involve the key update phase for all nonrevoked users interacting with the authority. Although it has the above advantage, in contrast, its disadvantage is that it requires the sender to possess the current revocation list whose management could be also a troublesome task. Recently, several attribute revocable ABE schemes [9, 45–47] that used the direct mode have been proposed.

For KP-ABE, a direct revocation method is, however, not possible yet for the normal present form of the KP-ABE algorithm since a normal KP-ABE scheme allows the sender only to specify attribute set associated to the ciphertext. A directly revocable KP-ABE scheme was first mentioned by Staddon et al. [66], but their scheme only works when the number of attributes associated with a ciphertext is exactly half of the size of the universe of real attributes.

And, for CP-ABE, such direct revocation can be done by using Ostrovsky et al.'s [9] scheme that supports negative clauses. To do so, one just adds conjunctively the AND of negation of revoked user identities (where each is considered as an attribute here). However, this solution is still somewhat low in efficiency. Because in this scheme, the ciphertext overhead scales with *O*(|*R*|) and the secret key overhead scales with *O*(log⁡ *n*) where *n* is the maximum size of revoked attributes set *R*.

Attrapadung and Imai [45] suggested a user-revocable ABE scheme by combining broadcast encryption schemes with ABE schemes. However, the data owner should take full charge of maintaining all the membership lists for each attribute group to enable the direct user revocation. This scheme is not applicable to the data outsourcing architecture, because the data owner will no longer be directly in control of data distribution after outsourcing their data to the external data server.

Liang et al. [46] proposed a CP-ABE scheme with efficient revocation. Their construction uses linear secret sharing and binary tree techniques, and can be proved secure in the standard model. In addition to the attribute set, each user is also assigned a unique identifier. Therefore, a user can be easily revoked by using his/her unique identifier.

All the above schemes [9, 45, 46] support user revocation, but they have no effect on attribute revocation. Recently, Wu and Zhang [47] first formalized the notion of adaptively secure ABE scheme supporting attribute revocation under direct revocation mode.

### 5.3. Hybrid Revocation Method

Combining the best advantages of both indirect and direct methods, Attrapadung and Imai [48] put forward the first hybrid revocable ABE scheme (HR-ABE) that allows a sender Alice to be able to select whether to use either direct or indirect revocation mode when encrypting a message. An HR-ABE scheme works as follows. When Alice selects the direct mode, she will specify the revocation list *R* directly into the encryption algorithm. And, when selecting the indirect mode, she is required only to specify the present time slot *t*. A user Bob has one secret key. Let *A* be the access policy associated to Bob's secret key. In addition, his secret key will be associated with a unique serial number ID. If ciphertext was from the direct mode, one can decrypt it solely by his key. If ciphertext was from indirect mode, he must obtain an update key from the authority at time *t*. Let *ω* be the attribute set associated with ciphertext. In this case, he can decrypt the ciphertext if *ω* satisfies *A*, and ID ∉ *R*. Notice that in the latter case, the authority specifies *R* when creating the update key and hence enforces revocation indirectly. This method supports user revocation, but it is unable to achieve attribute revocation. And the utilization of two subsystems increases the user's secret key in length.

So far, we showed and discussed revocable ABE schemes which are realized in two different ways. Both of them have advantages and disadvantages. For future work, the efficiency of the proposed schemes should be improved in shortening the secret key in length, reducing the update information published in quantity, and improving encryption and decryption algorithm in efficiency.

## 6. Accountable ABE

The ABE mechanism is a highly promising tool for secure fine-grained access control. For the purpose of secure access control, there is, however, still a critical functionality missing in the existing ABE schemes to prevent from key abuse. In particular, two problems of key abuse are extremely important in an ABE-based access control system: (i) illegal key sharing among colluding users and (ii) misbehavior of the semitrusted attribute authority including illegal key (re-)distribution.

To make the problems more concrete, in this section, we focus on the prevention of key abuse in ABE. At present, accountable ABE schemes can be divided into two kinds: accountable CP-ABE schemes [49–51] and accountable KP-ABE schemes [52, 53].

### 6.1. Accountable CP-ABE

The notion of accountable CP-ABE (CP-A^2^BE, in short) was first proposed by Li et al. [49] to address the key abuse problem existing in access control based on ABE. In the CP-A^2^BE scheme, user accountability is achieved by embedding additional user-specific information in the secret key. It can prevent sharing keys among users based on the following observation: The user's secret key consists of the attribute secret key and the user's identity. Therefore, if the user shares his secret key, the identity will be detected from the pirated device. The CP-A^2^BE scheme assumes that the key in a pirated device has a format specification, so it can only do white box tracking. In addition, it can only support operation between attributes and has a limited ability to express strategies. What is more, the public key certificate center is responsible for issuing certificates for all users, which has a serious impact on performance.

Li et al. [50] prevented illegal key sharing among users by proposing the notion of accountable and anonymous CP-ABE (CP-A^3^BE), firstly. This idea is achieved by binding user identity in the attribute secret key. In the proposed CP-A^3^BE scheme, user accountability can be achieved in black-box model by embedding additional user-specific information into the attribute secret key issued to that user, while still maintaining hidden access policy. But the disadvantage is that it increases the length of the decryption key and ciphertext.

Li et al. [51] proposed an accountable multiauthority CP-ABE scheme, which allows tracing the identity of a misbehaving user who leaks the decryption key to others and reduces the trust assumptions on not only the authorities but also the users. The tracing process is efficient because it has a lower computational cost compared with the existing accountable ABE schemes.

### 6.2. Accountable KP-ABE

The KP-ABE scheme is a promising cryptographic primitive which enables fine-grained access control over sensitive data. However, key abuse attacks in KP-ABE schemes may impede its wide applications especially in copyright-sensitive systems. To defend against this attack, Yu et al. [52] proposed an abuse free KP-ABE (AFKP-ABE) scheme by introducing hidden attributes such that the tracing algorithm can use them to identify any single piracy or partial colluding users. Their design enables black box tracing and does not require the well-formed secret key of the pirated device when compared with previous works. It is also efficient since the size of both the secret key and the ciphertext is *O*(log⁡ *N*), where *N* is the total number of users. This scheme is proved secure under the DBDH assumption and the D-linear assumption.

As a future work, one may focus on designing a tracing system to protect against arbitrary colluders. Recently, Wang et al. [53] first presented an accountable authority KP-ABE scheme which is proved secure under the modified Bilinear Decisional Diffie-Hellman (mBDDH) assumption in the standard model.

### 6.3. Comparison

A comparison of the CP-A^2^BE [49], CP-A^3^BE [50], and AFKP-ABE [52] schemes is given in Table 6, from which we can draw conclusions below. First, all of these three schemes can achieve user accountability. Second, although the CP-A^2^BE scheme achieves the authority accountability, it lacks feasibility by assuming a format specification of secret keys. And, third, both the CP-A^3^BE and the AFKP-ABE protect the sender's privacy, but the later can only partly hide attributes.

## 7. Attribute-Based Proxy Reencryption

To make data sharing more efficient, proxy reencryption (PRE) is proposed. Introduced by Mambo and Okamoto [67] and first defined by Blaze et al. [68], PRE extends the traditional public key encryption (PKE) to support the delegation of decryption rights. It allows a semitrusted party called proxy to transform a ciphertext encrypted under Alice's public key into another ciphertext of the same plaintext intended for Bob. The proxy, however, learns neither the decryption key nor the underlying plaintext. PRE is a useful cryptographic primitive and has many applications, such as secure distributed files systems [69] and email forwarding [68]. Considering an email forwarding scenario, Alice is going on vacation and wishes the others to be able to read the message in the encrypted email aiming to her. With a PRE scheme, she could fulfill this task without giving her secret key to either the mail server or Bob.

To date, PRE has been extended to adapt different cryptographic settings. In 2007, Green and Ateniese [70] extended the PRE technique in the identity-based cryptosystem and gave its applications. Meanwhile, another new notion was proposed in 2005, which is called the attribute-based cryptosystem [5]. However, the ABE scheme does not offer the capability of decryption to others when the user is offline. For this reason, the attribute-based PRE (ABPRE) scheme is proposed, which combines the traditional proxy reencryption with the ABE, so a user is able to empower designated users to decrypt the reencrypted ciphertext with the associated attributes of designated users.

Guo et al. [54] proposed the first attribute-based proxy reencryption scheme, but their scheme is based on key-policy and bidirectional. In 2009, Liang et al. [55] proposed the first ciphertext-policy attribute-based PRE (CP-ABPRE) scheme, in which a proxy is allowed to transform a ciphertext under a specified access policy (which is only represented as AND gates on positive and negative attributes) into the one under another access policy.

The previous ABPRE scheme demands a number of pairing operations that imply huge computational overhead. Based on Emura et al.'s [16] CP-ABE scheme which has a constant ciphertext length, Luo et al. [56] presented another ABPRE scheme with constant number of bilinear pairing operations. The computation cost and ciphertext length are reduced significantly compared to previous schemes.

In 2012, Seo and Kim [57] proposed a CP-ABPRE scheme which supports AND gates on multivalued and negative attributes. Compared with Liang et al.'s [55] scheme, Luo et al.'s have a new property named reencryption control which means the encryptor can decide whether the ciphertext can be reencrypted.

A CP-ABPRE scheme has many practical applications, such as fine-grained access control in cloud storage systems and medical records sharing among different hospitals. The aforementioned CP-ABPRE schemes, however, are only secure against CPA and support AND gates over attributes. The construction of a CCA secure CP-ABPRE scheme supporting any monotonic access policy remains unsolved. Liang et al. [58], for the first time, proposed a new single-hop unidirectional CP-ABPRE scheme, which supports attribute-based reencryption with any monotonic access structure, to tackle this problem. Despite being constructed in the random oracle model, it can be proved CCA secure under the decisional *q*-parallel BDHE assumption.

In 2013, Li presented a new ciphertext policy ABPRE scheme [59]. The ciphertext policy realized in his scheme is matrix access policy based on LSSS matrix access structure which is also used in Waters' CP-ABE scheme [13].

In future, we hope more and richer access policies such as hidden policies, tree policies, or access structures can be used in attribute-based PRE schemes. In addition, for the needs of practical applications, the efficiency of the schemes should be improved.

## 8. Future Work

The previous sections discuss the research process of ABE which has received considerable achievements. However, there still exist many problems worth further studying. According to application requirements and the shortcoming of the existing algorithms, some possible future works remain open and they are shown as follows.

1. Optimizing the construction method of CP-ABE schemes: it is known that the more complex an access structure is, the more complex a CP-ABE scheme will be and the more difficult it is to prove its security. Many existing construction methods add additional redundancy or restrictions (e.g., an attribute cannot repeatedly appear in the access structure), so it is necessary to optimize them. One solution is that we can try to design a new access structure which can be expressed in terms of monotone boolean formula and realized by an LSSS matrix whose size is as small as possible.
2. Improving the efficiency of attribute-based encryption schemes: almost all of the existing ABE schemes take bilinear pairings as a convenient construction way. But bilinear pairing has a higher computational complexity, which makes algorithms inefficient to some extent. Reducing the number of bilinear pairing operations will be a meaningful work. We can construct schemes where ciphertexts can be decrypted with a constant number of pairings by mathematics method. Or even we can also try not to use bilinear pairings in the design of the ABE algorithm (see next item).
3. Trying to build an ABE scheme by other technologies: identity-based encryption schemes can be built with the help of three theories, including bilinear pairings, quadratic residue, and lattice. ABE is widely considered to be a generalization and an expansion of IBE, but it is only built by the bilinear pairings which have limitations in terms of efficiency. So the research which uses lattice [20] or quadratic residue theory to build an ABE scheme is obviously a very meaningful work.
4. Accountable ABE: accountability can be a very good solution to prevent key abuse and key cloning. However, the existing accountable ABE schemes are only proved to be secure in the selective model. For further study, under three assumptions of the subgroup decision problem for 3 primes (3P-SDP) [19], it is necessary to design a high-efficiency accountable ABE scheme which can be proved to be full (adaptive) secure by using the dual system encryption method.
5. Focusing on the applicable and practicable research of ABE: ABE was initially put forward to achieve data confidentially and fine-gained access control. Then, it has been considered as the suitable cryptographic technology for the cloud environment. So on the basis of solving efficiency drawbacks, combined with technologies including PRE, anonymous authentication, access control, and keyword search, it is meaningful to propose more practical ABE schemes in cloud environment. ABE has received considerable achievements at the theoretical level, but unfortunately, it has not been widely used in practical applications. So we can expect that attribute-based cryptosystem and its applications will continue to be a research hot spot in the next few years.

The above is some possible future works of ABE and, certainly, there may be other problems which have been pointed out.

## 9. Conclusion

In recent years, attribute-based encryption is a relatively attractive research topic and has many attracting properties. It provides a fine-grained and noninteractive access control mechanism of encrypted data and has great potential applications in many fields. In this paper, firstly, we expound the emergence and development of ABE schemes. Then, we pay attention to main research directions of ABE, including multiauthority, use/attribute revocation, accountability, and proxy reencryption. Finally, we point out some possible future works of attribute-based encryption.

## Figures and Tables

**Table 1:** Comparison of security proof and policy complexity in different CP-ABE schemes.

Scheme	Access structure	Assumption	Model	Supported policy
Cheung and Newport [14]	AND gate between two-value attributes	DBDH	Selective	And, non
Nishide et al.'s [15]	AND gate among multivalue attributes	DBDH, D-linear	Selective	And
Emura et al.'s [16]	AND gate among multivalue attributes	DBDH	Selective	And
Bethencourt et al.'s [8]	Tree without bound	Generic group	Adaptive	And, or, threshold
Ibraimi et al.'s [18]	Tree without bound	DBDH	Selective	And, or, threshold
Goyal et al.'s [12]	Bounded tree	DBDH	Selective	Bounded and, or, threshold
Liang et al.'s [17]	Bounded tree	DBDH	Selective	Bounded and, or, threshold
Waters' [13]	LSSS matrix	DPBDHE	Selective	And, or, threshold
Lewko et al.'s [19]	LSSS matrix	3P-SDP	Adaptive	And, or, threshold

**Table 2:** Comparison of size of keys and ciphertext in different CP-ABE schemes.

Scheme	PK	MK	SK	Ciphertext
Cheung and Newport [14]	(3*n* + 1)*L*_*G*_1__ + *L*_*G*_2__	(3*n* + 1)*L*_*Zq*_	(2*n* + 1)*L*_*G*_1__	(*n* + 1)*L*_*G*_1__ + *L*_*G*_2__
Nishide et al.'s [15]	(2*N*′ + 1)*L*_*G*_1__ + *L*_*G*_2__	(2*N*′ + 1)*L*_*Zq*_	(3*n* + 1)*L*_*G*_1__	(2*N*′ + 1)*L*_*G*_1__ + *L*_*G*_2__
Emura et al.'s [16]	(*N*′ + 2)*L*_*G*_1__ + *L*_*G*_2__	(*N*′ + 1)*L*_*Zq*_	2*L*_*G*_1__	2*L*_*G*_1__ + *L*_*G*_2__
Bethencourt et al.'s [8]	3*L*_*G*_1__ + *L*_*G*_2__	*L*_*Zq*_ + *L*_*G*_1__	(2|*A*_*U*_| + 1)*L*_*G*_1__	(2|*A*_*U*_| + 1)*L*_*G*_1__ + *L*_*G*_2__
Ibraimi et al.'s [18]	(*n* + 1)*L*_*G*_1__ + *L*_*G*_2__	(*n* + 1)*L*_*Zq*_	(|*A*_*U*_| + 1)*L*_*G*_1__	(|*A*_*U*_| + 1)*L*_*G*_1__ + *L*_*G*_2__
Waters' [13]	(*n* + 2)*L*_*G*_1__ + *L*_*G*_2__	*L*_*G*_1__	(|*A*_*U*_| + 2)*L*_*G*_1__	(2|*A*_*U*_| + 1)*L*_*G*_1__ + *L*_*G*_2__
Lewko et al.'s [19]	(*n* + 2)*L*_*G*_1__ + *L*_*G*_2__	*L*_*Zq*_ + *L*_*G*_1__	(|*A*_*U*_| + 2)*L*_*G*_1__	(2|*A*_*U*_| + 1)*L*_*G*_1__ + *L*_*G*_2__

**Table 3:** Comparison of computational overhead in different CP-ABE schemes.

Scheme	Encryption	Decryption
Cheung and Newport [14]	(*n* + 1)*G*_1_ + 2*G*_2_	(*n* + 1)*C*_*e*_ + (*n* + 1)*G*_2_
Nishide et al.'s [15]	(2*N*′ + 1)*G*_1_ + 2*G*_2_	(3*n* + 1)*C*_*e*_ + (3*n* + 1)*G*_2_
Emura et al.'s [16]	(*n* + 1)*G*_1_ + 2*G*_2_	2*C*_*e*_ + 2*G*_2_
Bethencourt et al.'s [8]	(2|*A*_*C*_| + 1)*G*_1_ + 2*G*_2_	2|*A*_*U*_|*C*_*e*_ + (2|*S*| + 2)*G*_2_
Ibraimi et al.'s [18]	(|*A*_*C*_| + 1)*G*_1_ + 2*G*_2_	(|ω′| + 1)*C*_*e*_ + (|ω′| + 1)*G*_2_
Waters' [13]	(4|*A*_*C*_| + 1)*G*_1_ + 2*G*_2_	2|*A*_*U*_|*C*_*e*_ + 3|*A*_*U*_|*G*_2_
Lewko et al.'s [19]	(4|*A*_*C*_| + 1)*G*_1_ + 2*G*_2_	2|*A*_*U*_|*C*_*e*_ + 3|*A*_*U*_|*G*_2_

*A*_*c*_: attributes of ciphertext *C*; *A*_*u*_: attribute of user *u*.

|∗|: Number of element in ∗; *C*_*e*_: *e* operation, where *e* denotes bilinear paring.

*G*_*i*_: Group or operation in group, *i* = 1 or 2; *g* is a random generator of *G*.

*S*: Least interior nodes satisfying an access structure (include root node).

L*: Bit length of element in ∗; *n*: number of attributes in systems.

*N*′ = ∑_*i*=1_^*n*^ *n*_*i*_: Total number of possible value of attributes, where *n*_*i*_ is the number of possible values for attribute *i*.

**Table 4:** Comparison of central authority, security model, and type and length of ciphertext.

Scheme	Central authority	Security model	Prevent decryption by individual authorities	KP/CP-ABE	Length of ciphertext
Chase's [31]	Y	Selective-set	N	KP-ABE	(|*A*_*C*_| + 1)*L*_*G*_1__ + *L*_*G*_2__
Liu et al.'s [36]	Multiple	Full-security	Y	CP-ABE	(2|*A*_*C*_| + 1)*L*_*G*_1__ + *L*_*G*_2__
Lin et al.'s [32]	N	Selective-set	Y	FIBE	|*A*_*C*_|*L*_*G*_1__ + *L*_*G*_2__
Chase and Chow [34]	N	Selective-set	Y	KP-ABE	(|*A*_*C*_| + 1)*L*_*G*_1__ + *L*_*G*_2__
Lekwo and Waters [35]	N	Full-security	Partially	CP-ABE	2|*A*_*C*_|*L*_*G*_1__ + (|*A*_*C*_| + 1)*L*_*G*_2__
Han et al.'s [37]	N	Selective-set	Y	KP-ABE	(|*A*_*C*_| + 2)*L*_*G*_1__ + *L*_*G*_2__

**Table 5:** Comparison of computing cost.

Schemes	Authority setup	KeyGen	Encryption	Decryption
Chase's [31]	(|*U*| + 1)*E*	(|*A*_*U*_| + 1)*E*	(|*A*_*C*_| + 2)*E*	|*A*_*C*_|*E* + (|*A*_*C*_| + 1)*P*
Liu et al.'s [36]	(|*U*| + *N*)*E*	(4*d* + |*A*_*U*_|)*E* + |*I*_*U*_|*E*	(3|*A*_*C*_| + 2)*E*	(|*A*_*C*_| + 1)*E* + 2|*A*_*C*_|*P*
Chase and Chow [34]	(|*U*| + 2*N*)*E*	(|*U*| + |*I*_*U*_|^2^)*E*	(|*A*_*C*_| + 2)*E*	|*A*_*C*_|*E* + (|*A*_*C*_| + 1)*P*
Lekwo and Waters [35]	2*NE*	2|*A*_*U*_|*E*	(5|*A*_*C*_| + 1)*E*	3|*A*_*C*_|(*E* + *P*)
Han et al.'s [37]	(|*U*| + 2*N*)*E*	(|*A*_*U*_| + 3|*I*_*U*_|)*E*	(|*A*_*C*_| + 3)*E*	|*A*_*C*_|*E* + (|*A*_*C*_| + |*I*_*C*_| + 1)*P*

**Table 6:** Comparison of CP-A^2^BE, CP-A^3^BE, and AFKP-ABE.

Scheme	Trace property	Trace effect	Sender hides	Assumption	Supported policy
CP-A^2^BE [49]	White box	Authority, user	Null	DBDH, CDH	And
CP-A^3^BE [50]	Black box	User	Policy	DBDH, D-linear	And
AFKP-ABE [52]	Black box	User	Part attributes	DBDH, D-linear	And, or, threshold
